# Tenascin-C expression is significantly associated with the progression and prognosis in gastric GISTs

**DOI:** 10.1097/MD.0000000000014045

**Published:** 2019-01-11

**Authors:** Chaoyong Shen, Chengshi Wang, Yuan Yin, Huijiao Chen, Xiaonan Yin, Zhaolun Cai, Zhixin Chen, Bo Zhang, Zongguang Zhou

**Affiliations:** a Department of Gastrointestinal Surgery; b West China Clinical Research Center of Breast Disease; c Department of Pathology; d Institute of Digestive Surgery and State Key Laboratory of Biotherapy, West China Hospital, Sichuan University, Chengdu, Sichuan, China.

**Keywords:** gastrointestinal stromal tumors, prognosis, progression, Tenascin-C

## Abstract

Tenascin-C (TNC), an extracellular matrix glycoprotein, has been implicated in progression of various types of cancer. However, few reports exist on TNC expression in gastrointestinal stromal tumors (GISTs). We here attempted to investigate the expression pattern and prognostic significance of TNC in gastric GISTs. We studied TNC expression in 122 gastric GISTs tissue samples by immunohistochemistry, and examined the correlations of TNC expression with clinicopathological parameters and survival of gastric GISTs. The TNC-high expression was observed in 30 (24.6%) of 122 of gastric GISTs. The high levels of TNC expression in gastric GISTs was significantly associated with tumor size (*P* < .001), multivisceral resection (*P* = .006), metastasis at initial diagnosis (*P* = .006), mitotic count (*P* = .002) and NIH risk classification (*P* = .015). The TNC mRNA and protein levels were found to significantly downregulated in tumors without progression compared to those tumors which occurred tumor progression during the follow-up period (*P* < .05). As for the prognostic analysis, it revealed that tumor size, mitotic count, surgical margins, multivisceral resection, and TNC expression were independent predictors of PFS for gastric GISTs (*P* < .05). The overexpression of TNC may be as a possible marker for the metastatic potential of gastric GISTs patients.

## 1 Introduction

Gastrointestinal stromal tumors (GISTs), which continue to increase in frequency, are the most common mesenchymal tumors in the alimentary tract.^[1,2]^ GISTs are well known to metastasize to the distant tissues/organs, and this aggressiveness can also be observed in the low-risk tumors.^[3,4]^ Hepatic metastasis, accounts for approximately 20% to 60% of all distant metastases, is the most feared complication by far and the major cause of death for GISTs patients.^[5,6]^ Previously, several risk-stratification schemes, which merely based on clinicopathological features, have been proposed to evaluate malignant potential of GISTs;^[7–9]^ but molecular events regarding its malignancy have rarely been explored so far. Thus, a better understanding of the molecular mechanism responsible for GISTs progression has been anticipated.

The invasive aspects of tumor progression represent complex molecular event, which highly depends on the interactions between tumors cells and a considerable great number of extracellular matrix (ECM) components. Although previous studies have shown that ECM may play an essential role in many cancer's metastasis/progression and offer novel opportunities to prevent and treat cancers,^[10–12]^ the role of ECM proteins on the progression of patients with GISTs remains unknown and need to be clarified. Tenascin-C (TNC), a glycoprotein of the ECM, which is secreted from both tumor cells and myofibroblasts and can directly or indirectly affect the tumor invasiveness.^[13]^ TNC is absent or generally repressed in most adult tissues, while a striking upregulation is observed in some pathological conditions such as wound healing, inflammation and in a variety of neoplasias.^[14,15]^ Continuously growing evidence suggests that high TNC is associated with poor prognosis in colorectal, breast, lung, esophageal and prostate cancer.^[16–20]^ In addition, Wiksten et al reported that the cumulative 5-year survival in patients with strong TNC expression was 42% compared to 26% in those with negative-to-moderate expression in gastric cancer.^[21]^

But little is known about the expression and critical role of TNC in the development and progression of GISTs as yet. Therefore, on the basis of data obtained from 122 consecutive patients with gastric GISTs in our institution, we attempted to investigate the relationship between clinicopathological parameters and TNC expression. Additionally, we also further explored its potential value as a prognostic marker in gastric GISTs patients.

## 2 Materials and methods

### 2.1 Patient collection

A total of 122 gastric GISTs patients were consecutively involved at the West China Hospital, Sichuan University from January 2011 to November 2014. The inclusion criteria for specimen were performed as follows: all primary gastric GISTs were confirmed by pathological examinations; patients underwent surgical resection; patients with complete medical records and all tumor samples were KIT (CD117) positive. The exclusion criteria for specimen were taken as follows: patients received preoperative imatinib/sunitinib therapy prior to surgery; patients underwent exploratory operation alone; patients who had other malignant digestive tumors. The clinicopathological parameters, including follow-up data, age at diagnosis, gender, hospital stay, tumor size, mitotic count, clinical symptom, distant metastasis, and NIH risk classifications^[8]^ were carefully reviewed. The Institutional Review Board of West China Hospital, Sichuan University approved the study protocol and all patients provided written informed consent according to institutional guidelines.

### 2.2 Immunohistochemical staining procedure

The archival formalin-fixed, paraffin-embedded tissues of gastric GISTs were obtained from Department of Pathology at our institution, and cut into 3 to 4 μm serial sections. The anti-human TNC mouse monoclonal antibody [D87] was purchased from Abcam (Ab86182, Cambridge, UK). Specimens were deparaffinized with xylene and rehydrated in decreasing concentrations of alcohols to water. Endogenous peroxidase was blocked using hydrogen peroxide for 20 min. Slides were heated at 120°C in 10 mM citrate buffer (pH 6.0) for 5 min and cooled to room temperature. The slides were incubated with appropriate antibody overnight at 4°C at the following dilution: TNC, 1:300. Sections were incubated with anti-mouse/rabbit immunoglobulins (Abcam, Cambridge, UK) for 30 min at 37°C. The immunohistochemical reactions were visualized with the avidin–biotin–peroxidase complex, and then DAB-chromogen substrate mixture (DAKO) was applied. Finally, sections were counterstained with hematoxylin, dehydrated, cleared mounted under cover slips. The immunohistochemistry staining was performed at the same time and the scoring was independently recorded by 2 seasoned researchers. According to the proportion and intensity of positive-staining cells, immunohistochemical scores were measured.^[20]^ The mean percentage of positive tumor cells was assigned from 0 to 100% (<10%, 0; 10∼25%, 1; 26∼50%, 2; 51∼75%, 3; ≥76%, 4). Omission of the primary antibody was considered as negative control while similar treating process of IHC in hepatocellular carcinoma was utilized as positive control.^[22]^ And staining intensity was scored as follows: negative 0; weak 1; moderate 2; and intense 3, and mean staining intensity obtained from five independent fields was calculated as: (negative)% × 0 + (weak)% × 1 + (moderate)% × 2 + (intensive)% × 3. The low expression was defined as ≤ 1.5, whereas the slides were scored 1.5-3 as high expression.

### 2.3 Real-time quantitative PCR

Total RNA was extracted from fresh-frozen gastric GISTs tissues by using the Trizol reagent (Invitrogen, CA, USA) according to the protocol of manufacturer. A NanoDrop ND-1000 spectrophotometer was utilized to determine the concentration and purity of isolated RNA. Real-time quantitative PCR reaction was performed using SYBR Premix Ex Taq kit (Takara, Kyoto, Japan) as described by the manufacturer. β-actin was used as an endogenous control. Primers were obtained from Invitrogen. The primers used for detection of TNC mRNA were 5’-CTGAAGGTGG AGGGGTACAG-3’ (F) and 5’-AGAAGGATCTGCCATTGTGG-3’ (R), while for β-actin the primers were 5’GTGGCCGAGGACTTTGATTG3’ (F) and 5’CCTGTAACAACGCATCTCATATT3’ (R). All samples were run in triplicate, and the quantification of TNC was normalized to β-actin expression using the 2-ΔΔCt method.

### 2.4 Western blot analysis

Total cellular protein was extracted from fresh-frozen tumor tissue specimens with a RIPA Lysis Buffer (Beyotime, China) containing protease inhibitor, phenylmethylsulfonyl fluoride and phosphatase for 20 min, and followed by centrifugation at 14,000 g for 15 min at 4°C. Equal amounts of tissue lysate per lane were loaded onto 20% sodium dodecyl sulfate polyacrylamide gels. And proteins were then transferred to PVDF membranes (Millipore, Bedford, USA), and blocked with 5% skimmed milk under ambient temperature for 1 h. Membranes were incubated with anti-human TNC mouse monoclonal antibody (1:1200 dilution, Abcam, Cambridge, UK) at 4°C overnight, and which were subsequently incubated with the horseradish peroxidase-conjugated anti-goat second antibody (Invitrogen) at a dilution of 1:5000 for 2 h at room temperature. Imaging was performed with a ChemiDocTM XRS+System (BIO-RAD), and quantification was conducted using Image software. β-actin was used as loading control for normalization.

### 2.5 Statistical analysis and follow-up

Calculations statistical analysis was performed with the Statistical Package for the Social Science (SPSS), version 21.0 for Windows (SPSS Inc, Chicago, IL). Measurement data were expressed as mean ± standard deviation and enumeration data were described as percentage. Associations between discrete variables were analyzed using χ^2^ test or Fisher exact test. Cumulative survival was determined using the Kaplan-Meier method and the log-rank test was used to determine the statistical significance. Progression-free survival (PFS) was defined from the start of any treatment until disease progression. The univariate and multivariate analyses were used to explore independent prognostic values by Cox regression. Differences with 2-sided *P* < .05 indicated statistical significance. Hazard ratios and 95% confidence intervals were assessed for each factor. Follow-ups were carried out by office visit, telephone call, or outpatient clinic visit from November 2017 to December 2017. Abdominal ultrasonography and CT, blood biochemistry, and evaluation of liver and kidney functions were also performed.

## 3 Results

### 3.1 Patient characteristics

Data of 122 eligible patients with primary gastric GISTs were collected, including 56 males (45.8%) and 66 females (54.1%), with the male-to-female ratio is 0.85:1. The median age at initial diagnosis was 59 years (range: 30∼84 years), with a mean age of 56.97 ± 11.35 years. The average size for the entire cohort 7.12 ± 5.40 cm with a median of 6.0 cm (1.0∼35.0 cm). In patients reporting main symptoms upon initial presentation, 50 patients exhibited abdominal distention/discomfort/pain, 34 cases with alimentary tract hemorrhage, 27 patients were asymptomatic. A total of 114 patients underwent laparotomy, while 8 patients underwent endoscopic/laparoscopic surgery (4 cases with endoscopic resection). Radical resection (R0) was performed in 117 cases. In terms of NIH classification, very low-, low-, intermediate- and high-risk of recurrence were detected in 7(5.7%), 26(21.3%), 33(27.0%) and 56(45.9%) patients, respectively. Of these patients, 14 patients had hepatic and/or abdominal cavity metastasis. A total of 19 patients received tyrosine kinase inhibitor as adjuvant therapy, with a median time medication of 21 months (12∼46 months).

### 3.2 Associations of TNC expression with clinicopathological parameters

**Figure 1:**
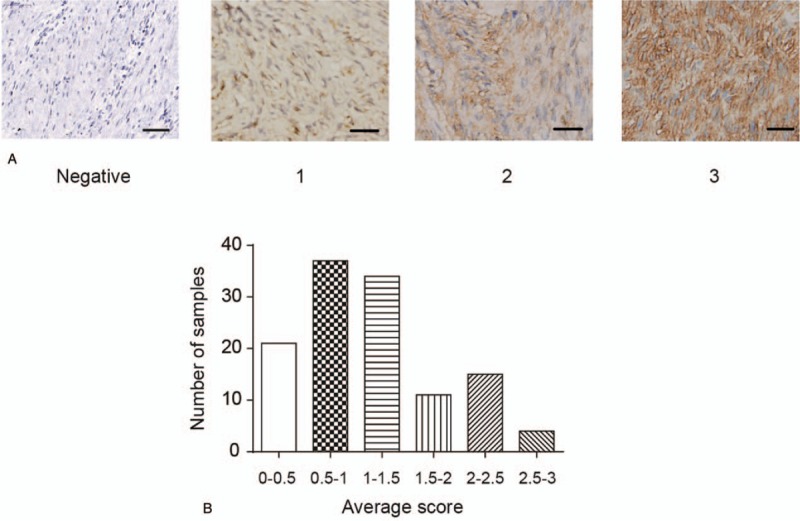
Representative immunohistochemical staining of TNC in gastric GISTs. A: Expression patterns for TNC immunohistochemistry in GISTs were shown. B: Diagram were drawn to show the distribution of IHC scores. GISTs = gastrointestinal stromal tumors, IHC = immunohistochemistry, TNC = Tenascin-C.

To investigate TNC expression in gastric GISTs, we immunohistochemically evaluated a panel of 122 specimens. Positive features of TNC expression were mainly localized in the cytoplasm of tumor cells and extracellular matrix (Fig. 1). In most samples, GIST samples were recorded with epithelioid cell morphology composed of round cells with eosinophilic cytoplasm arranged in sheets. And TNC-high expression was observed in 30 (24.6%) of 122 of gastric GISTs. The mean size of TNC-high tumors was significantly larger than that of TNC-low tumors (10.86 ± 8.05 vs 5.90 ± 3.47 cm, respectively; *P* < .001). We found that TNC expression in gastric GISTs was significantly associated with multivisceral resection (*P* = .006), metastasis at initial diagnosis (*P* = .006), mitotic count (*P* = .002) and NIH risk classification (*P* = .015). However, there were no correlation between TNC expression and gender, age, hospital stay, surgical margins, co-morbidity and tumor location. The associations between the TNC expression and clinicopathological factors are summarized in Table 1.

**Table 1:**
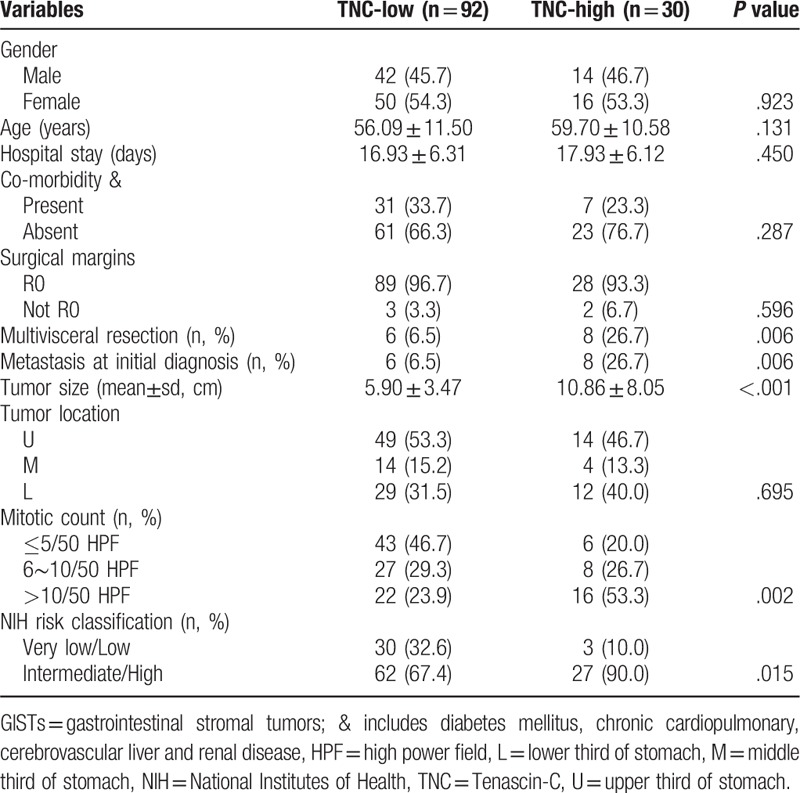
Comparison of clinicopathologic characteristics according to TNC expression in gastric GISTs (n = 122).

### 3.3 TNC expression increased in Gastric GISTs with progression

**Figure 2:**
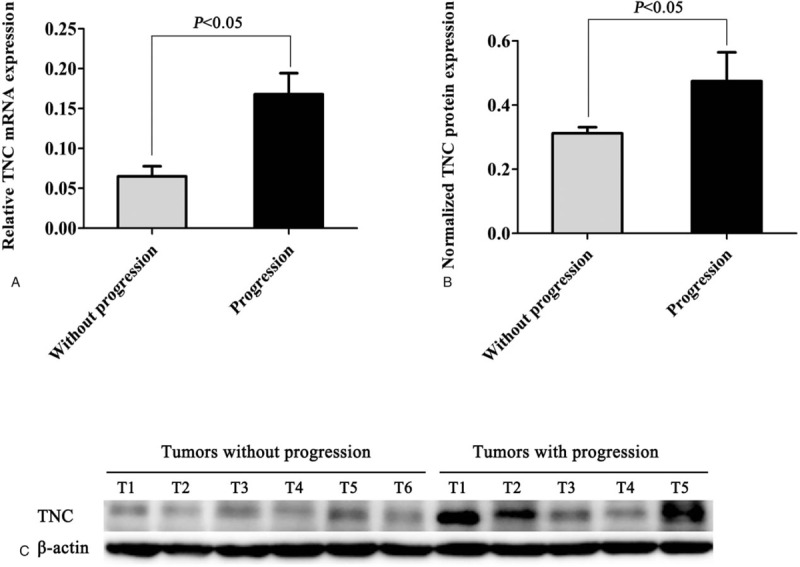
The protein expression of TNC was determined by RT-qPCR and western blot with β-actin as a loading control. The TNC mRNA (A) and protein levels (B, C) were found to significantly downregulated in tumors without progression compared to those tumors which occurred tumor progression during the follow-up period (*P* < .05). TNC = Tenascin-C.

We applied both RT-qPCR and western blot methods to further explore the expression pattern of TNC between tumors without progression and tumors with progression in the high-risk tumor tissues. As shown in Figure 2, the TNC mRNA and protein levels were found to significantly downregulated in tumors without progression compared to those tumors which occurred tumor progression during the follow-up period (*P* < .05). In the limited samples, the expression level of TNC obtained by western blot was in consistency with the staining score by IHC method. These findings suggested that TNC might play a crucial role in the development and progression of gastric GISTs.

### 3.4 Survival outcomes and prognostic factors

**Figure 3:**
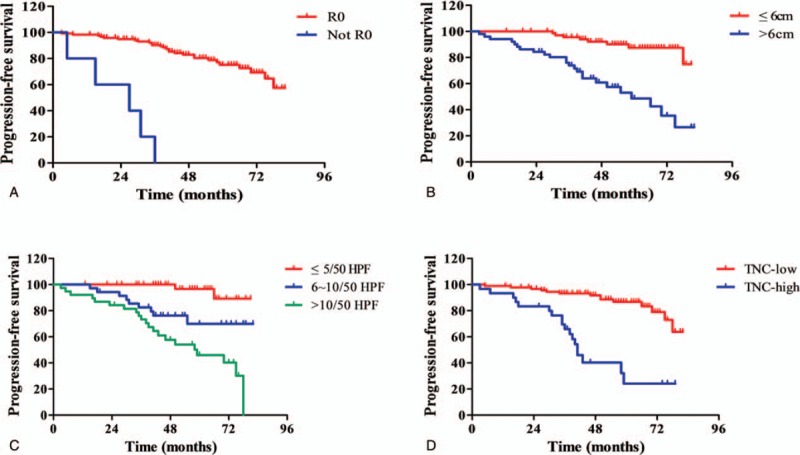
Kaplan-Meier survival curves of progression-free survival in patients with primary gastric GIST (n=122). A: Patients with R0 resection showed a better PFS than that who did not achieve R0 resection (*P* < .001); B: Comparison of progression-free survival between tumors with ≤6 cm and >6 cm (*P* < .001); C: The tumors with mitotic count ≤5/50HPF showed significant better PFS compared with those of 6∼10/50HPF and >10/50HPF (*P* < .001); D: A worse prognosis was noted in patients with TNC-high expression in comparison to those with TNC-low expression (*P* < .001). GISTs = gastrointestinal stromal tumors , PFS = progression-free survival, TNC = Tenascin-C.

By the end of the follow-up period (November 2017 to December 2017), a total of 32 patients experienced tumor progression, with a median progression-free survival time of 50 months (range: 3∼82 months). Survival curves were drawn between TNC-low and TNC-high gastric GISTs (Fig. 3). The TNC-low group had a significantly better PFS than those in patients with TNC-high at 5-year (86.7% vs 24.1%, respectively; *P* < .001). Univariate analysis of prognostic factors through the Kaplan–Meier method showed that PFS was statistically correlated with tumor size (*P* < .001), mitotic count (*P* < .001), metastasis at initial diagnosis (*P* < .001), NIH risk classification (*P* = .001), surgical margins (*P* < .001), multivisceral resection (*P* < .001), and TNC expression (*P* < .001). By incorporating these parameters into the Cox multivariate regression proportional hazards model, it revealed that tumor size, mitotic count, surgical margins, multivisceral resection, and TNC expression were independent predictors of PFS for gastric GISTs (Table 2).

**Table 2:**
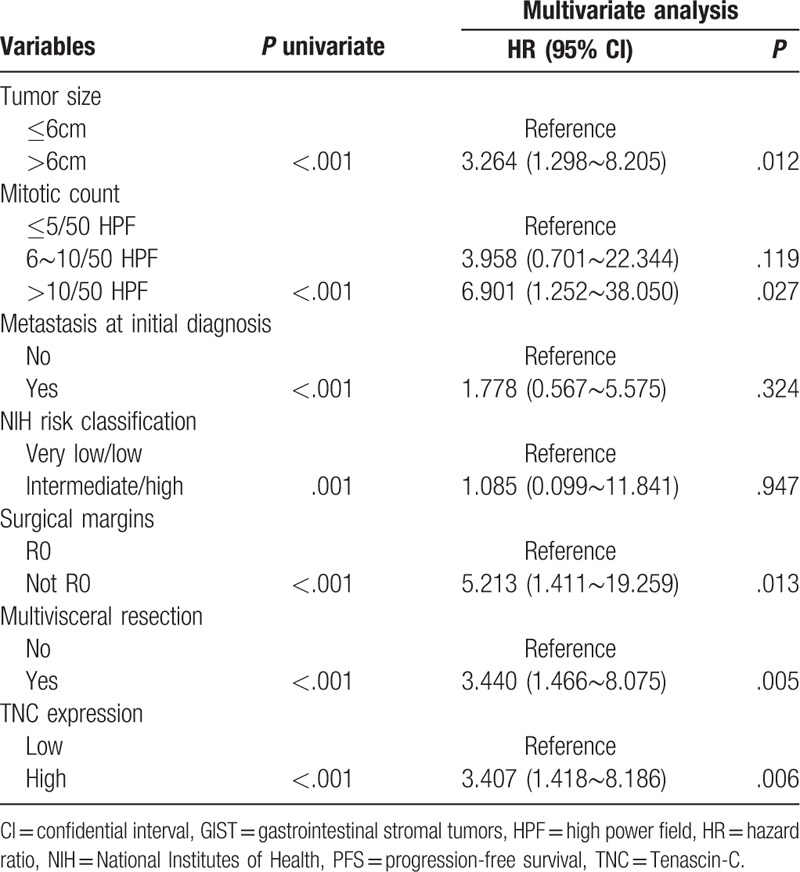
Univariate and multivariate analyses for prognostic parameters with PFS in gastric GISTs using Cox proportional hazards regression modeling.

## 4 Discussion

Nowadays, surgery is the mainstay treatment for GISTs patients. Unfortunately, approximately 30% to 50% of GISTs who underwent radical resection may experience tumor progression within 2 years postoperatively, especially those with tumors size >10 cm^[23–25]^. Numerous studies have demonstrated that the tumor microenvironment is an emerging source of novel therapeutic targets in cancer. As such, to improve the unfavorable long-term outcome of GISTs, it is essential to explore the expression pattern of invasion related ECM molecules of this disease. In this work, we first studied TNC expression in 122 gastric GISTs tissue samples by immunohistochemistry, and examined the correlations of TNC expression with clinicopathological parameters and survival of gastric GISTs. We found that the TNC-low group had a significantly better PFS than those in patients with TNC-high at 5-year, and TNC expression were independent predictors of PFS for gastric GISTs. The expression of TNC might be a clinically effective parameter for distinguishing malignancy of gastric GISTs.

TNC, which is composed of 6 monomers linked at their N-termini with disulfide bonds, is a large hexameric extracellular matrix protein that involved in tumor growth, migration, metastasis, tumorigenesis and immunosuppression.^[14,20,26]^ TNC is transiently expressed in many developing organs or under tissue regeneration, but it may maintain sustained TNC activity in tumors. Maseruka et al reported that TNC levels are markedly reduced after wound healing is completed and TNC expression is virtually absent in avascular scar tissue. TNC facilitates tumor malignization as high TNC expression has been associated with worsened prognosis in various tumors.^[16–20,26,27]^ The 5-year metastasis-free rate in patients with TNC-positive was significantly lower than that in those with TNC-negative for clear cell renal carcinoma with stage 1–3.^[27]^ Our study have found that TNC-high in gastric GISTs is associated with a poor prognosis in accordance with their findings. However, the prognostic significance of TNC expression remains controversial. Yang et al found that breast ductal carcinoma patients with positive expression of TNC has a shortened overall survival compared to the negative group,^[19]^ while diametrically opposing conclusions were drawn by Shoji and colleagues.^[28]^ This phenomenon may attribute to different sources of TNC because a functional difference in TNC may exist between the cancer cells and stromal cells.^[20]^ In this study, we found that positive features of TNC expression were mainly localized in the cytoplasm of tumor cells and extracellular matrix.

TNC has a pleiotropic role in advancing metastasis by promoting cancer cell motility and invasion.^[29]^ Hirata et al have reported that the shRNA-mediated knockdown of endogenous tenascin-C does not affect proliferation of glioblastoma cells, but it abolishes cell migration on a 2-dimensional substrate and tumor invasion with brain tissue changes in a xenograft model.^[30]^ Epithelial mesenchymal transition (EMT) represents conversion of an epithelial cells to mesenchymal cells, which also participates in the process of wound healing/fibrosis and malignant tumors.^[31]^ Recent study has proved a negative prognostic role of the EMT-related markers (CD44, SLUG, N-cadherin and VSIG1/Glycoprotein A34) in gastrointestinal stromal tumors.^[32]^ Previously, another study has shown that TNC efficiently induces pancreatic cancer cells to undergo epithelial-mesenchymal transition progression, regulates focal adhesion formation via JNK/c-Jun signaling activation, by which tumor cell invasion and migration was enhanced.^[33]^ In addition, Yang et al^[34]^ has also reported that TNC may promote EMT-like change and proliferation, which lead to poor prognosis for patients with colorectal cancer. From the above statement, we believe that the aberrant expression of molecules is involved in the process of EMT in GISTs, and TNC was also proved in carcinomas to be involved in EMT. However, whether TNC overexpression promotes GISTs progression by induction of EMT or not is unclear so far. Thus, the detailed mechanism of TNC involving in GISTs progression should be explored in further study, which is also the major limitation of this study.

Several factors have been reported as prognostic indications, such as tumor location and size, mitotic index and body mass index.^[5,6,13]^ In the present cohort of primary gastric GISTs, the tumor size, mitotic count, surgical margins, multivisceral resection, as well as the TNC expression were the independent predictors of PFS for gastric GISTs, which is in agreement with their findings. As for prognostic value of TNC expression, many studies have also shown that TNC can be as a prognostic marker in various cancers.^[18–20]^ Indeed, to explore the molecules which are involved in the process of metastasis might provide additional information on the nature of tumor malignization, and a novel therapeutic target for GISTs can be finally found.

## 5 Conclusions

In conclusion, the collective findings from our study show for the first time that the TNC-low group had a significantly better PFS than those in patients with TNC-high at 5-year (86.7% vs 24.1%), and the overexpression of TNC may be as a possible marker for the metastatic potential of gastric GISTs patients. Additionally, this study also sets the stage for further investigations on the basic mechanisms that underlie GISTs metastasis.

## Acknowledgments

The authors gratefully acknowledge the whole staff of the Department of Gastrointestinal Surgery, West China Hospital, who generously provided assistance in the collection of data throughout the duration of the study.

## Author contributions

**Conceptualization:** Bo Zhang.

**Data curation:** Chaoyong Shen.

**Formal analysis:** Chengshi Wang.

**Funding acquisition:** Bo Zhang.

**Investigation:** Huijiao Chen, Xiaonan Yin.

**Methodology:** Chengshi Wang, Huijiao Chen.

**Project administration:** Xiaonan Yin, Zhixin Chen.

**Resources:** Yuan Yin.

**Software:** Yuan Yin, Xiaonan Yin, Zhaolun Cai.

**Supervision:** Zhixin Chen.

**Validation:** Zhaolun Cai, Bo Zhang, Zongguang Zhou.

**Visualization:** Zhaolun Cai.

**Writing – original draft:** Chaoyong Shen.

**Writing – review & editing:** Bo Zhang, Zongguang Zhou.

Bo Zhang orcid: 0000-0002-0254-5843.
